# Abediterol, a novel long-acting β_2_-agonist: bronchodilation, safety, tolerability and pharmacokinetic results from a single-dose, dose-ranging, active-comparator study in patients with COPD

**DOI:** 10.1186/s12890-016-0266-5

**Published:** 2016-07-20

**Authors:** Jutta Beier, Helena Pujol, Beatriz Seoane, Eulalia Jimenez, Carol Astbury, Eric Massana, Sandrine Ruiz, Gonzalo de Miquel

**Affiliations:** insaf Respiratory Research Institute, Biebricher Allee 34, 65187 Wiesbaden, Germany; AstraZeneca, Barcelona, Spain; Almirall R&D Centre, Barcelona, Spain

**Keywords:** COPD, LABA, Bronchodilation, Chronic respiratory disease

## Abstract

**Background:**

Abediterol is a novel, once-daily long-acting β_2_-agonist in development for the treatment of chronic obstructive pulmonary disease (COPD) and asthma in combination with an anti-inflammatory agent. This Phase IIa, randomised, double-blind, crossover study investigated the bronchodilation, safety, tolerability and pharmacokinetics of abediterol in patients with moderate to severe COPD.

**Methods:**

Seventy patients (aged ≥40 years, Global initiative for chronic Obstructive Lung Disease Stage II/III) were randomised (1:1:1:1:1:1) to single doses of abediterol 0.625, 2.5, 5 or 10 μg, indacaterol 150 μg or placebo. Spirometry was performed up to 36 h post-dose. Pharmacokinetics were assessed in a subset of patients (*N* = 20). Safety and tolerability were evaluated throughout the study.

**Results:**

Abediterol (all doses) significantly improved change from baseline in trough forced expiratory volume in 1 s (FEV_1_) compared with placebo (0.102, 0.203, 0.233 and 0.259 L for abediterol 0.625, 2.5, 5 and 10 μg, respectively; all *p* < 0.0001; primary endpoint). Abediterol 2.5, 5 and 10 μg significantly improved trough FEV_1_ compared with indacaterol 150 μg (0.092, 0.122 and 0.148 L, respectively; all *p* < 0.0001). Improvements in bronchodilation were maintained at all time points post-dose versus placebo (all abediterol doses) and from 15 or 30 min post-dose versus indacaterol 150 μg with abediterol 2.5, 5 and 10 μg (all *p* < 0.05). Abediterol had low systemic exposure; incidence of treatment-emergent adverse events was similar between treatment groups.

**Conclusions:**

All doses of abediterol (0.625–10 μg) provided clinically and statistically significant, dose-dependent improvements in bronchodilation versus placebo, and abediterol 2.5, 5 and 10 μg gave significant improvements versus indacaterol. All doses of abediterol were safe and well tolerated in patients with COPD.

**Trial registration:**

Clinicaltrials.gov NCT01425814. Registered 29 August 2011.

## Background

Inhaled bronchodilator medications – anticholinergics and β_2_-agonists – are central to the symptomatic treatment of chronic obstructive pulmonary disease (COPD) [1]. Two classes of long-acting bronchodilators are currently available: (1) long-acting muscarinic antagonists (LAMAs), i.e. tiotropium, glycopyrrolate and umeclidinium (all once-daily [QD] administration), and aclidinium bromide (twice-daily [BID] administration); and (2) long-acting β_2_-agonists (LABAs), which include formoterol BID, salmeterol BID, indacaterol QD and olodaterol QD. In addition, four LABA/LAMA combinations have recently become available for the management of COPD, namely QD indacaterol/glycopyrrolate (QVA149), vilanterol/umeclidinium, tiotropium/olodaterol and twice-daily aclidinium/formoterol [2–5].

Long-acting bronchodilators are currently the mainstay of maintenance therapy for patients with moderate to severe COPD [1, 6]. However, the addition of inhaled corticosteroids (ICS) may be of benefit to some patients, particularly those with a history of exacerbations and more severe disease, as recommended in the Global initiative for chronic Obstructive Lung Disease (GOLD) guidelines [6, 7]. Combination therapies including both a LABA and an ICS in a single inhaler are more effective at reducing the frequency of moderate to severe exacerbations than either drug administered individually [1, 6].

Abediterol is a new LABA being developed as a combination therapy with an anti-inflammatory agent for the treatment of both asthma and COPD [1, 8]. As a new chemical entity, studies of abediterol as monotherapy have been conducted to establish its clinical efficacy and safety profile [9–15]. In vitro pre-clinical studies with human β_2_-adrenoreceptor over-expressing cells in isolated guinea pig tissues and in vivo animal models have demonstrated that abediterol displays superior bronchodilatory potency and similar or superior selectivity for β_2_-adrenoreceptors over β_1_-adrenoreceptors compared with formoterol, indacaterol, salmeterol, vilanterol and olodaterol. In vivo models have also confirmed that abediterol has a duration of action similar or superior to LABA reference compounds, whilst demonstrating a reduced effect on heart rate [9, 16–19]. In early-phase clinical trials, abediterol was associated with rapid and sustained improvements in bronchodilation compared with placebo, and doses ≤10 μg were found to be safe and well tolerated in healthy subjects and patients with asthma or COPD, with a safety profile consistent with that expected for the drug class [12, 14, 15, 20].

Here, we report the results of a Phase IIa single-dose study designed to investigate the bronchodilation, safety, tolerability and pharmacokinetics (PK) of four doses of abediterol (0.625, 2.5, 5 and 10 μg) compared with placebo and with indacaterol 150 μg in patients with moderate to severe COPD. In addition to the evaluation of standard bronchodilation parameters such as forced expiratory volume in 1 s (FEV_1_) and forced vital capacity (FVC), inspiratory capacity (IC) was also investigated as an indirect measure of hyperinflation, a key aspect of COPD which causes dyspnoea, limits exercise capacity and contributes to reduced quality of life [21–23].

## Methods

### Patients

Male and female patients aged ≥40 years with a smoking history ≥10 pack-years and moderate to severe clinically stable COPD were eligible for inclusion in the study. Patients had post-salbutamol FEV_1_ ≥30 % and <80 % of the predicted normal value (based on European Community for Steel and Coal predicted values) and post-salbutamol FEV_1_/FVC ratio <70 %. Exclusion criteria included asthma (as defined by the Global Initiative for Asthma [24]); oxygen therapy for ≥15 h/day; respiratory tract infection or COPD exacerbation within 6 weeks of the screening visit; hospitalisation for COPD exacerbation ≤3 months before screening; body mass index ≥40 kg/m^2^; hypertension (≥160/100 mmHg) or resting heart rate ≥100 bpm at screening; and any clinically significant respiratory or cardiovascular condition. Patients with QT values at screening of ≥500 ms or QT interval corrected using Bazett’s formula values >450 (male) or >470 (female) ms were excluded. Patients with a known hypersensitivity to β_2_-adrenergic agonists, inhaled medication or drugs chemically related to abediterol were also excluded.

Concomitant use of anticholinergic agents, short-acting β_2_-agonists (except inhaled salbutamol as relief medication), LABAs, methylxanthines, cromolyn sodium, nedocromil, leukotriene modifiers, non-selective β_1_-blocking agents (selective β_1_-agents were permitted if stable ≥4 weeks prior to screening), roflumilast or β_2_-antagonists (including eye drops) was not permitted during the study. Prohibited concomitant medications were to be withdrawn before the patient entered the study and discontinued prior to screening as follows: long-acting inhaled anticholinergic agents, ≥72 h; short-acting inhaled anticholinergic agents, ≥12 h; other oral, intranasal or parenteral anticholinergic agents, ≥72 h; oral short-acting β_2_-agonists, ≥24 h; inhaled long-acting β_2_-agonists, 48 h; continuous oral or parenteral corticosteroids, ≥4 weeks; short-term oral or parenteral corticosteroids to treat exacerbations, ≥6 weeks (or 3 months if exacerbation led to hospitalisation); methylxanthines, ≥48 h; cromolyn sodium or nedocromil, ≥5 days; leukotriene modifiers, ≥48 h; non-selective β_1_ blocking agents, ≥2 weeks; any over-the-counter medicinal product or herbal product which could have had an effect on any efficacy or safety assessment, ≥36 h, except paracetamol; any other investigational drug ≥1 month or the equivalent of 6 half-lives of the treatment; roflumilast, ≥1 week. Oral corticosteroids were permitted if used at stable doses equivalent to ≤10 mg/day of prednisone. ICS were allowed if stable ≥4 weeks prior to screening and throughout the study (including the day of study visits) provided dosage/regimen remained constant during the study.

### Study design and treatment

This was a randomised, double-blind, double-dummy, placebo- and active-controlled, single-dose, six-way crossover Phase IIa study, conducted at eight centres in Germany, to assess the efficacy, safety and tolerability of abediterol in patients with moderate to severe COPD. The PK of abediterol was also assessed in a subset of patients.

**Fig. 1:**
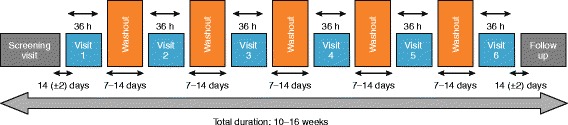
Study design

After a 14-day run-in period to assess clinical stability, patients were assigned to one of six treatment sequences according to a William’s design for crossover studies and using a balanced 1:1:1:1:1:1 randomisation ratio. Each treatment visit lasted 36 h and was separated by a 7- to 14-day washout period. A follow-up assessment was performed 14 days after the final treatment period (Fig. 1). On the first morning of each treatment visit, patients received a single dose of abediterol (0.625, 2.5, 5 or 10 μg), indacaterol 150 μg (active comparator) or placebo. Administration of abediterol took place at 09:00 ± 1:00 h each morning by inhalation from the Genuair® device, while indacaterol was administered by inhalation from the Onbrez® Breezhaler®. To ensure blinding, placebo could be delivered via either device. At each visit, patients used both devices (always Genuair® first) with one or both delivering placebo. The external appearances of both active and placebo abediterol devices (Genuair®) were identical, the only difference between them was the active ingredient. With indacaterol, however, there were minor differences in the appearance of the capsules compared with those of placebo. Therefore, in order to ensure blinding of indacaterol was maintained for both patients and investigators, independent personnel at each centre were responsible for placing the capsule in the chamber of the Breezhaler® device prior to dosing.

The study complied with the declaration of Helsinki and the International Conference on Harmonisation and Good Clinical Practice guidelines. The protocol was approved by an independent ethics committee (Ethikkommission der, Landesärztekammer Hessen, Im Vogelsgesang 3, 60488 Frankfurt am Main). All patients provided written informed consent.

### Efficacy assessments

All efficacy endpoints are reported for the intent-to-treat (ITT) population, defined as all randomised patients who received ≥1 dose of abediterol and had FEV_1_ values for baseline and ≥1 post-dose treatment period.

The primary efficacy variable was the change from baseline in trough FEV_1_ on Day 2 (23–24 h post-dose on Day 1). Key secondary efficacy variables included: change from baseline in trough FVC at Day 2; change from baseline in normalised FEV_1_ and FVC area under the curve (AUC) for 0–12, 12–24 and 0–24 h post-dose; change from baseline and absolute values for FEV_1_, FVC and IC at all time points; peak and time to peak FEV_1_ and FVC (maximum value observed post-dose) on Day 1. Normalised AUC values were calculated using the trapezoidal method, dividing the pulmonary function value by the corresponding time intervals. Bronchial reversibility was assessed 10–15 mins post-administration of 4 × 100 μg puffs of salbutamol and was defined as ≥12 % and ≥200 mL change from pre-test FEV_1_. All measures of pulmonary function were obtained using spirometers provided by eResearch Technology (ERT) GmbH (Estenfeld, Germany) and met American Thoracic Society and European Respiratory Society recommendations for accuracy and precision. IC was calculated as the highest value of three acceptable readings at each scheduled time point. Spirometry data were manually reviewed throughout the study by an independent, blinded, spirometric expert from a centralised spirometry company (ERTcare GmbH).

### Pharmacokinetics

The PK sub-study was performed on the per-protocol population, defined as a subset of the ITT population who had not presented serious deviations from the protocol that may have affected efficacy (e.g., met all inclusion/exclusion criteria liable to affect the efficacy assessment). PK parameters were determined for each dose of abediterol from plasma samples collected up to 24 h post-dose in a subset of 20 patients. Abediterol plasma levels were determined using a validated liquid chromatography with tandem mass spectrometry assay with a lower limit of quantification of 0.05 pg/mL. Concentrations of abediterol in human plasma were evaluated using a non-compartmental approach by least squares (LS) linear regression analysis of the terminal phase of the semilogarithmic concentration-time curve. PK parameters were calculated according to standard methods using Kinetica software, version 4.2 (Thermo Scientific, USA). The following PK parameters were calculated: maximum plasma concentration (C_max_), time to reach maximum plasma concentration (t_max_), terminal elimination half-life (t_1/2_), area under the plasma concentration-time curve from zero to the last quantifiable time point (AUC_0-t_), area under the plasma concentration-time curve from zero to infinity (AUC_0-∞_), total body clearance from plasma (CL/f) and apparent volume of distribution (Vz/f).

### Safety and tolerability assessments

The safety population comprised all patients who received ≥1 dose of the study drug. Adverse events (AEs) were monitored and recorded throughout the study. Blood pressure, 12-lead electrocardiograms (ECG) and clinical laboratory tests (standard haematology, blood chemistry, urinalysis [dipstick], blood glucose and serum potassium) were recorded at screening and/or pre-dose and at multiple time points post-administration up to 36 h.

### Statistical methods

Planned enrolment was 60 patients. Allowing for a 15 % dropout rate (48 patients were planned to complete treatment), the study had 80 % power (two-sided test at 0.05 level) to detect a difference of 100 mL between active treatment and placebo for the primary efficacy variable assuming a standard deviation for within-patient differences of 240 mL.

Demographic and baseline characteristics were described for the safety population using descriptive statistics. Efficacy variables were analysed using an analysis of covariance (ANCOVA) model for crossover designs. Fixed-effect factors were sequence, treatment and period, the random effect was patient within sequence, and baseline FEV_1_ was a covariate. The patient effect was assumed to be random and therefore the covariance structure from the set of measurements from one patient was the compound symmetry. Between-treatment comparisons were conducted by means of contrasts on the treatment factor. LS means methodology was used to estimate differences between treatments. Two-sided hypothesis tests with a significance level of 0.05 were used for all statistical comparisons of active treatments versus placebo. As this was an exploratory study, the Type I error rate was not adjusted for multiple treatment comparisons.

The analysis of dose proportionality for AUC_0-t_ and C_max_ parameters was performed by means of a fixed-effect model, which was carried out in an exploratory manner. Data were analysed using Statistical Analysis Software (SAS Institute Inc., Cary, NC, USA) version 9.1.3.

## Results

### Patient disposition

**Fig. 2:**
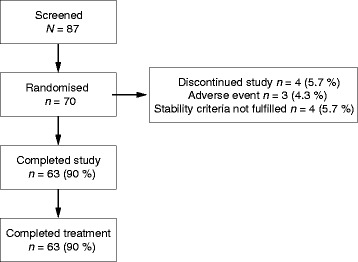
Patient disposition

Of 87 patients who underwent screening, 70 were randomised (44 male, 26 female) to treatment and included in the ITT and safety populations, and 63 completed the study (Fig. 2). Discontinuations were due to AEs (*n* = 3) or stability criteria not being met (*n* = 4). Demographic and baseline characteristics data are summarised in Table 1. Patients were 42–79 years old (mean 61.2 years), with a mean smoking history of 48.96 pack-years; 58.6 % (41/70) were current smokers. Overall, 70 % (49/70) of patients had GOLD Stage II (moderate) disease and 30 % (21/70) had GOLD Stage III (severe) COPD. Mean post-bronchodilator FEV_1_ was 1.72 L (58.03 % of predicted FEV_1_). Approximately 95 % of patients used concomitant medication during the study: 31.3–35.3 % used a concomitant ICS (budesonide, 17.9–20.6 % and fluticasone propionate, 13.4–15.2 %) and 81.8–83.8 % used inhaled salbutamol. Other common drug classes included antithrombotic agents (22.1–22.7 %) and angiotensin-converting enzyme inhibitors (19.1–21.2 %).

**Table 1:** Patient demographics and baseline characteristics (safety population)

Characteristic	Patients (*N* = 70)	Median	Quartiles	
			Q1	Q3
Age (years), mean (SD)	61.2 (7.7)	62.0	56.0	66.0
BMI (kg/m^2^), mean (SD)	27.35 (4.20)	27.21	24.25	30.12
Gender (male), n (%)	44 (62.86)			
Race (Caucasian), n (%)	69 (98.57)			
Current smoker, n (%)	41 (58.57)			
Smoking history (pack-years), mean (SD)	48.96 (27.98)	41.25	31.00	61.20
Severity of airflow limitation, n (%)				
Moderate (GOLD Stage II)	49 (70.0)			
Severe (GOLD Stage III)	21 (30.0)			
Post-bronchodilator FEV_1_ (L)				
Mean (SD)	1.72 (0.57)	1.71	1.20	2.04
% predicted, mean (SD)	58.03 (12.57)	57.95	49.20	68.90
Post-bronchodilator FVC (L)				
Mean (SD)	3.57 (0.99)	3.52	2.76	4.23
% predicted, mean (SD)	97.64 (16.62)	98.70	86.50	107.80
FEV_1_/FVC ratio (%), mean (SD)	48.53 (10.28)	48.10	40.50	56.30
Bronchial reversibility (%), mean (SD)^a^	15.74 (13.25)	13.25	6.90	20.20
Prior COPD medication^b^, n (%)				
SABA	66 (94.29)			
LAMA	17 (24.29)			
LABA/ICS	17 (24.29)			
LABA	16 (22.86)			
ICS	8 (11.43)			
SABA + SAMA	4 (5.71)			
Xanthines	4 (5.71)			
SAMA	2 (2.86)			
Influenza vaccine	1 (1.43)			
Oxygen	1 (1.43)			

^a^Bronchial reversibility was assessed using salbutamol 100 μg per puff and was defined as ≥12 % and ≥200 mL change from pre-test FEV_1_.’ *BMI* body mass index, *COPD* chronic obstructive pulmonary disease, *FEV*_*1*_ forced expiratory volume in 1 s, *FVC* forced vital capacity, *GOLD* Global initiative for chronic Obstructive Lung Disease, *ICS* inhaled corticosteroid, *LABA* long-acting β_2_-agonist, *LAMA* long-acting muscarinic antagonist, *SABA* short-acting β_2_-agonist, *SAMA* short-acting muscarinic antagonist, *SD* standard deviation

^b^Prior medication defined as any medication within 15 days prior to the date of informed consent and up to the first investigational medicinal product administration

### Efficacy

#### Primary efficacy variable

**Fig. 3:**
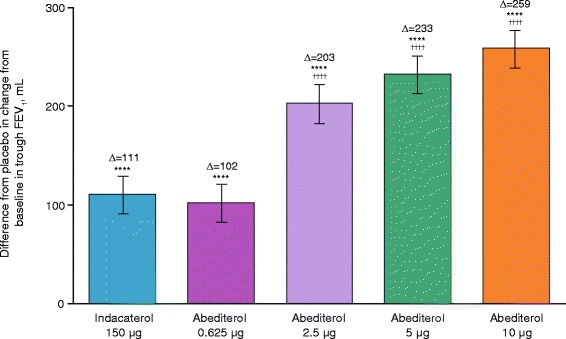
Change from baseline in trough FEV_1_ at Day 2 (per-protocol population). ****p < 0.0001 vs placebo; ^††††^ *p* < 0.0001 vs indacaterol. Data reported as LS mean difference from placebo (ANCOVA) ± SE. ANCOVA, analysis of covariance; FEV_1_, forced expiratory volume in 1 s; LS, least squares; SE, standard error

All doses of abediterol showed significant improvements from baseline in mean trough FEV_1_ 23–24 h post-dose compared with placebo. LS mean differences were 0.102, 0.203, 0.233 and 0.259 L for abediterol 0.625, 2.5, 5 and 10 μg, respectively, and 0.111 L for indacaterol 150 μg (all *p* < 0.0001; Fig. 3). Additionally, abediterol 2.5, 5 and 10 μg achieved significantly greater improvements from baseline in trough FEV_1_ compared with indacaterol 150 μg (LS mean differences 0.092, 0.122 and 0.148 L for 2.5, 5 and 10 μg abediterol, respectively; *p* < 0.0001), while abediterol 0.625 μg was broadly equivalent to indacaterol 150 μg (Fig. 3).

#### Secondary efficacy variables

**Fig. 4:**
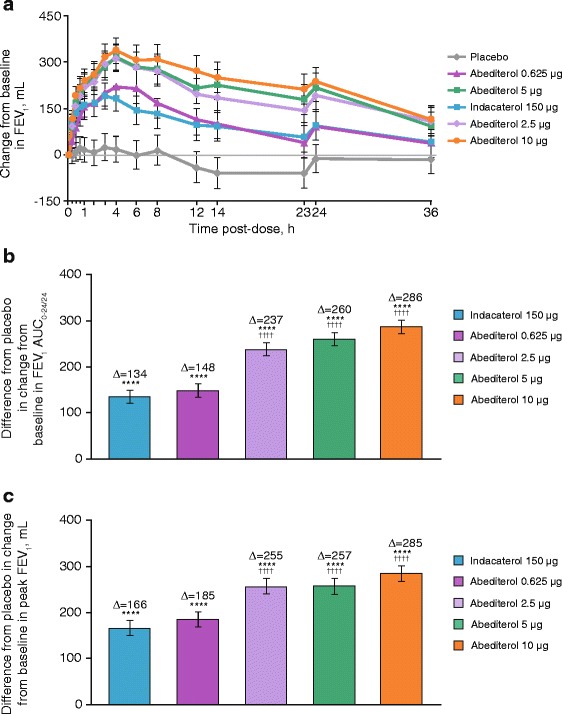
**a** Change from baseline in FEV_1_ over time. Data reported as LS mean ± SE; *p* < 0.05 abediterol (all doses) vs placebo, all time points; *p* < 0.05 abediterol 2.5–10 μg vs indacaterol, all time points from 0.5 h post-dose. **b** Placebo-subtracted change from baseline in FEV_1_ AUC_0–24_. **c** Placebo-subtracted change from baseline in peak FEV_1_ (per-protocol population). **a** *p* < 0.05 abediterol (all doses) vs placebo, all time points; *p* < 0.05 abediterol 2.5–10 μg vs indacaterol, all time points from 0.5 h post-dose. Data reported as LS means with 95 % CIs (ANCOVA). **b** ****p < 0.0001 vs placebo; ^††††^ *p* < 0.0001 vs indacaterol. Data reported as LS mean difference from placebo (ANCOVA) ± SE. **c** ****p < 0.0001 vs placebo, ^††††^ *p* < 0.0001 vs indacaterol. Data reported as LS mean difference from placebo (ANCOVA) ± SE. ANCOVA, analysis of covariance; AUC_0-24/24_, area under the curve over the 24 h period immediately after morning IMP administration; CI, confidence interval; FEV_1_, forced expiratory volume in 1 s; IMP, investigational medicinal product; LS, least squares; SE, standard error

**Fig. 5:**
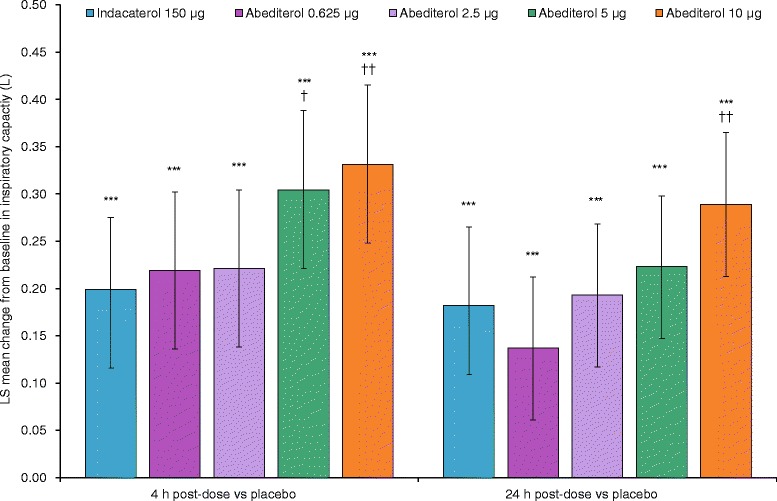
Change from baseline in inspiratory capacity versus placebo and indacaterol (ITT population). Data reported as LS mean difference ± 95 % CI. ***p < 0.001 vs placebo; ^†^ *p* < 0.05 vs indacaterol; ^††^ *p* < 0.01 vs indacaterol. CI, confidence interval; LS, least squares

Increases from baseline in mean FEV_1_ occurred with all doses of abediterol compared with placebo at all time points on Days 1 and 2 (*p* < 0.05) and from 15 or 30 min post-dose compared with indacaterol (Fig. 4a–c). The median time to peak FEV_1_ was 3–4 h post-administration for all doses of abediterol and 3 h for indacaterol; however, peak FEV_1_ was of greater magnitude with abediterol 2.5, 5 and 10 μg than with indacaterol (LS mean treatment difference of 0.089, 0.091 and 0.119 L, respectively; *p* < 0.0001). Improvements in FEV_1_ AUC_0-12_, AUC_12-24_ and AUC_0-24_ were observed with abediterol compared with placebo (*p* < 0.0001, all doses) and indacaterol (*p* < 0.0001, all doses ≥2.5 μg; Table 2). Furthermore, clinically meaningful increases from baseline in mean FEV_1_ occurred with all doses of abediterol compared with placebo at all time points on Days 1 and 2 (*p* < 0.05), and from 15 or 30 min post-dose versus indacaterol 150 μg for the 2.5, 5 and 10 μg dose levels. Changes from baseline in trough FVC at Day 2 were also greater with abediterol compared with placebo (LS mean differences of 0.146, 0.259, 0.294 and 0.333 L for 0.625, 2.5, 5 and 10 μg abediterol, respectively; *p* ≤ 0.0002) and compared with indacaterol (LS mean differences 0.092 L [*p* = 0.0176], 0.129 L [*p* = 0.0009] and 0.166 L [*p* < 0.0001] for abediterol 2.5, 5 and 10 μg, respectively). In addition, improvements in FVC AUC_0–12,_ AUC_12–24_ and AUC_0–24_ were recorded for all doses of abediterol compared with placebo (*p* < 0.0001) and for abediterol 2.5, 5 and 10 μg compared with indacaterol (*p* ≤ 0.033; Table 3). Similarly, improvements in change from baseline in IC versus placebo and indacaterol 150 μg were recorded with all doses of abediterol at 4 and 24 h post-administration (4 h, *p* ≤ 0.0001; 24 h, *p* ≤ 0.0004 vs placebo; *p* ≤ 0.0054 at both 4 and 24 h vs indacaterol; Fig. 5).

**Table 2:** LS mean differences in changes from baseline in peak FEV_1_ and FEV_1_ AUC outcomes (ITT population)

FEV_1_ variable (L)	Comparison	Abediterol 0.625 μg (*N* = 67)			Abediterol 2.5 μg (*N* = 66)			Abediterol 5 μg (*N* = 66)			Abediterol 10 μg (*N* = 67)		
		LS mean	95 % CI	*p*-value	LS mean	95 % CI	*p*-value	LS mean	95 % CI	*p*-value	LS mean	95 % CI	*p*-value
Normalised AUC_0-12_ at Day 1	vs placebo	0.164	0.134, 0.195	<0.0001	0.246	0.216, 0.277	<0.0001	0.255	0.224, 0.285	<0.0001	0.283	0.252, 0.313	<0.0001
	vs indacaterol	0.027	−0.004, 0.057	0.0826	0.109	0.079, 0.139	<0.0001	0.117	0.087, 0.148	<0.0001	0.145	0.115, 0.176	<0.0001
Normalised AUC_12-24_ at Day 1	vs placebo	0.131	0.095, 0.167	<0.0001	0.227	0.192, 0.263	<0.0001	0.264	0.228, 0.299	<0.0001	0.290	0.254, 0.326	<0.0001
	vs indacaterol	−0.002	−0.038, 0.035	0.9296	0.095	0.058, 0.131	<0.0001	0.131	0.095, 0.167	<0.0001	0.157	0.121, 0.193	<0.0001
Normalised AUC_0-24_ at Day 1	vs placebo	0.148	0.118, 0.179	<0.0001	0.237	0.206, 0.268	<0.0001	0.260	0.229, 0.291	<0.0001	0.286	0.255, 0.317	<0.0001
	vs indacaterol	0.014	−0.017, 0.045	0.3799	0.102	0.071, 0.134	<0.0001	0.125	0.094, 0.157	<0.0001	0.152	0.120, 0.183	<0.0001
Peak FEV_1_ at Day 1	vs placebo	0.185	0.147, 0.223	<0.0001	0.255	0.217, 0.292	<0.0001	0.257	0.219, 0.295	<0.0001	0.285	0.247, 0.323	<0.0001
	vs indacaterol	0.019	−0.019, 0.057	0.3350	0.089	0.050, 0.127	<0.0001	0.091	0.053, 0.129	<0.0001	0.119	0.081, 0.157	<0.0001

Analyses were performed on the ITT population. For both placebo and indacaterol, *N* = 68

LS mean differences and *p*-values obtained from an ANCOVA model for crossover designs with change from baseline in FEV_1_ variable as response, sequence, treatment group and period as fixed effect factors, patient within sequence as a random effect and the corresponding baseline value at each period as a covariate

ANCOVA, analysis of covariance; *AUC*_*0-12*_ area under the curve over the 12 h period immediately after morning IMP administration, *AUC*_*12-24*_ area under the curve over the 12 h nighttime period after morning IMP administration, *AUC*_*0-24*_ area under the curve over the 24 h period immediately after morning IMP administration, *FEV*_*1*_ forced expiratory volume in 1 s, *IMP* investigational medicinal product, *ITT* intent-to-treat, *LS* least squares, *N* ITT population size of treatment group

**Table 3:** Peak FVC and FVC AUC outcomes (safety population)

FVC variable (L)	Comparison	LS mean differences in changes from baseline											
		Abediterol 0.625 μg (*N* = 67)			Abediterol 2.5 μg (*N* = 66)			Abediterol 5 μg (*N* = 66)			Abediterol 10 μg (*N* = 67)		
		LS mean	95 % CI	*p*- value	LS mean	95 % CI	*p*-value	LS mean	95 % CI	*p*-value	LS mean	95 % CI	*p*-value
Normalised AUC_0-12_ at Day 1	vs placebo	0.227	0.169, 0.284	<0.0001	0.280	0.222, 0.337	<0.0001	0.301	0.243, 0.358	<0.0001	0.347	0.289, 0.405	<0.0001
	vs indacaterol	0.010	−0.047, 0.068	0.7258	0.063	0.05, 0.121	0.0323	0.084	0.027, 0.142	0.0044	0.130	0.072, 0.188	<0.0001
Normalised AUC_12-24_ at Day 1	vs placebo	0.182	0.114, 0.249	<0.0001	0.308	0.201, 0.376	<0.0001	0.352	0.285, 0.420	<0.0001	0.386	0.319, 0.454	<0.0001
	vs indacaterol	−0.026	−0.094, 0.042	0.4471	0.100	0.031, 0.166	0.0045	0.144	0.076, 0.212	<0.0001	0.178	0.110, 0.247	<0.0001
Normalised AUC_0-24_ at Day 1	vs placebo	0.208	0.153, 0.263	<0.0001	0.294	0.239, 0.349	<0.0001	0.332	0.276, 0.387	<0.0001	0.369	0.313, 0.424	<0.0001
	vs indacaterol	−0.007	−0.063, 0.048	0.7996	0.079	0.023, 0.135	0.0058	0.117	0.061, 0.172	<0.0001	0.154	0.098, 0.209	<0.0001
Peak FVC at Day 1	vs placebo	0.152	0.035, 0.270	0.0110	0.191	0.074, 0.309	0.0015	0.201	0.083, 0.318	0.0009	0.244	0.127, 0.361	<0.0001
	vs indacaterol	0.013	−0.104, 0.131	0.8219	0.052	−0.065, 0.170	0.3829	0.062	−0.056, 0.179	0.3024	0.105	−0.012, 0.222	0.0795

Analyses were performed on the ITT population. For both placebo and indacaterol, *N* **=** 68

LS mean differences and *p*-values obtained from an ANCOVA model for crossover designs with change from baseline in FVC variable as response, sequence, treatment group and period as fixed effect factors, patient within sequence as a random effect and the corresponding baseline value at each period as a covariate

ANCOVA, analysis of covariance; *AUC*_*0-12*_ area under the curve over the 12 h period immediately after morning IMP administration, *AUC*_*12-24*_ area under the curve over the 12 h nighttime period immediately after morning IMP administration, *AUC*_*0-24*_ area under the curve over the 24 h period immediately after morning IMP administration, *CI* confidence interval, *FVC* forced vital capacity, *IMP* investigational medicinal product, *ITT* intent-to-treat, *LS* least squares, *N* ITT population size of treatment group

#### Pharmacokinetics

**Fig. 6:**
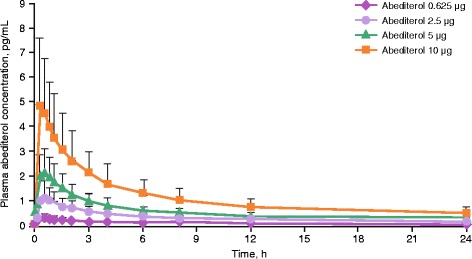
Plasma concentration of abediterol over time (PK population). Data reported as mean ± SD. PK, pharmacokinetic; SD, standard deviation

Abediterol was associated with very low systemic exposure and was quantifiable in the 24-hour profile for all doses (with the exception of the 12- and 24-hour time points for eight of 19 patients at the 0.625 μg dose). T_max_ occurred 0.5–1.5 h post-dose, with similar values across all doses (Fig. 6, Table 4). Mean elimination half-life values ranged between 15 and 27.5 h across doses. There were no relevant trends in the estimated half-lives, total body clearance from plasma or apparent volume of distribution during the terminal phase within the administered doses, overall suggesting a linear pharmacokinetic behaviour of abediterol. AUC_(0-t)_ and C_max_ values for abediterol increased proportionally to the administered dose within the dose range of 0.625–10 μg.

**Table 4:** Mean PK parameters of abediterol in patients with COPD

Parameter (unit)	Abediterol 0.625 μg		Abediterol 2.5 μg		Abediterol 5 μg		Abediterol 10 μg	
	(*n* = 19)		(*n* = 19)		(*n* = 19)		(*n* = 20)	
	mean	(SD)	mean	(SD)	mean	(SD)	mean	(SD)
C_max_ (pg/mL)	0.374	0.195	1.230	0.637	2.24	0.927	5.10	2.67
t_max_^a^ (h)	0.50	0.25–8	0.50	0.22–1.5	0.45	0.25–1.5	0.50	0.25–0.75
t_½_ (h)	27.5^b^	9.02	17.4^c^	6.76	16.0^d^	5.04	15.1^e^	3.35
AUC_0-t_ (pg.h/mL)	2.29	1.17	7.84	2.82	13.3	4.48	28.0	11.0
AUC (pg.h/mL)	5.95^b^	2.66	12.5^c^	6.01	19.7^d^	4.48	38.1^e^	14.1
CL/f (L/h)	119^b^	40	252^c^	135	268^d^	70.6	310^e^	161
V_z_/f (L)	4345^b^	753	5736^c^	2629	6219^d^	2675	7060^e^	5199

*AUC*_*0-t*_ area under the concentration-time curve from zero to the last quantifiable time point, *AUC* area under the concentration-time curve, *CL/f* total body clearance of drug from plasma after extravascular administration, *C*_*max*_ maximum measured plasma concentration, *COPD* chronic obstructive pulmonary disease, *h* hour(s), *n* number of patients with data, *PK* pharmacokinetic, *SD* standard deviation, *t*_*max*_ time to reach maximum concentration, *t*_*½*_ terminal elimination half-life, *V*_*z*_ */f* apparent volume of distribution during terminal phase after extravascular administration

^a^median value (min-max), ^b^ *n* = 5, ^c^ *n* = 11, ^d^ *n* = 12, ^e^ *n* = 9

### Safety and tolerability

All 70 patients received ≥1 dose of the study drug and were included in the safety analysis. Overall, abediterol was well tolerated and had a good safety profile. Thirty-two patients reported 57 treatment-emergent AEs (TEAEs): 37 were considered mild, 17 moderate and three severe (one event of COPD exacerbation following abediterol 5 μg and one event each of dizziness and vomiting in the same patient following abediterol 10 μg). Similar numbers of TEAEs occurred with each regimen, including placebo (Table 5). The most frequently reported TEAEs were nasopharyngitis (15 cases in 14 patients) and headache (eight cases in seven patients). Treatment-related TEAEs occurred in ≤3 % of patients for each dose and were similar to controls. No clinically meaningful dose-response relationship was observed for any safety outcomes. No deaths occurred during the study. Three patients discontinued due to TEAEs, one each following administration of abediterol 0.625 μg (nasopharyngitis), abediterol 5 μg (COPD exacerbation) and indacaterol 150 μg (dyspnoea). No clinically relevant changes in blood pressure, 12-lead ECG or clinical laboratory tests were observed in any treatment group during the course of the study.

**Table 5:** TEAEs occurring in ≥2 patients in any treatment group (safety population)

TEAE, n (%)	Number of patients (%) reporting TEAE						
	Placebo (*N* = 68)	Indacaterol 150 μg (*N* = 68)	Abediterol				Total (*N* = 70)
			0.625 μg (*N* = 67)	2.5 μg (*N* = 66)	5 μg (*N* = 66)	10 μg (*N* = 67)	
Any	9 (13.2)	10 (14.7)	7 (10.4)	5 (7.6)	6 (9.1)	9 (13.4)	32 (45.7)
Nasopharyngitis	2 (2.9)	5 (7.4)	4 (6.0)	1 (1.5)	1 (1.5)	2 (3.0)	15 (21.4)
Headache	3 (4.4)	0	1 (1.5)	0	2 (3.0)	2 (3.0)	8 (11.4)
Dyspnoea	0	2 (2.9)	0	0	0	0	2 (2.9)

*TEAE* treatment-emergent adverse event

## Discussion

Several Phase II clinical studies of the efficacy and safety of abediterol have been performed in patients with asthma [10–12, 14], however this was the first study to investigate this novel LABA in patients with COPD. The results demonstrate that single doses of abediterol 0.625, 2.5, 5 or 10 μg achieved a significant bronchodilatory response compared with placebo and were safe and well tolerated in patients with moderate to severe COPD.

Compared with placebo, all doses of abediterol resulted in clinically meaningful and significantly greater increases from baseline in peak and trough FEV_1_ and FVC, with an increasing response with increasing doses of abediterol. All doses of abediterol produced a change in trough FEV_1_ that exceeded the minimum clinically important difference of 100 mL versus placebo [25]. Similarly, abediterol produced dose-related improvements in IC versus placebo, suggesting that abediterol reduces lung hyperinflation. Taken together, these results suggest that abediterol is likely to be effective for the treatment of COPD and has a clear once-daily dosing profile.

Furthermore, abediterol 2.5, 5 and 10 μg achieved significantly greater improvements in bronchodilatory response compared with indacaterol 150 μg, starting from 30 min post-dose for the 2.5 μg dose and from 15 min post-dose for the abediterol 5 and 10 μg doses. These results are consistent with pre-clinical studies, which have shown that abediterol displays greater affinity for β_2_-adrenoceptors and a higher functional selectivity for β_2_-adrenoceptors over β_1_-adrenoceptors than indacaterol in a cellular model of overexpressed human receptors, and greater potency than indacaterol in isolated human bronchi [9]. Although this was only a single-dose investigation and the results need to be confirmed following repeat dosing, it could be hypothesised that the results may translate to a reduction in dynamic hyper-inflation which would result in an improvement in symptoms (breathlessness), an increase in exercise tolerance and a potential reduction in exacerbations (through raising a patient’s exacerbation reporting-threshold) [26].

Inhaled doses of abediterol 0.625–10 μg showed a linear dose-dependent relationship for PK parameters. High and aberrant physiological values for the total clearance from plasma and the apparent volume of distribution were observed, suggesting a very low bioavailability of abediterol. Plasma exposure obtained after single inhaled doses of 0.625–10 μg of abediterol was very low (sub-picogram range), thereby reducing the potential for undesired systemic side effects [27]. Abediterol was safe and well tolerated by patients with moderate to severe COPD, with no evidence that the increased bronchodilatory response compared with indacaterol was associated with an increase in the incidence of AEs.

Overall, the results reported here for abediterol in patients with COPD are consistent with the efficacy results for abediterol in patients with asthma, including studies using different inhaler devices [10–12, 14]. Abediterol has demonstrated good efficacy in patients with persistent stable asthma, with significant improvements in bronchodilation compared with placebo as early as 5 min post-dose [11, 12]. Studies of abediterol delivered via the Genuair® or Cyclohaler® devices have shown that peak FEV_1_ is reached 2–4 h post-dose, and clinically and statistically significant bronchodilation is maintained over 24 h post-dose [10–12, 14]. The results of these studies, together with the data we report here, are compatible with a once-daily dosing regimen in patients with COPD or asthma.

The statistical power calculation in this study was based on a comparison of abediterol with placebo, requiring 48 patients to complete the study to provide 80 % power to detect a treatment difference of 100 mL in trough FEV_1_ at Day 2. As the protocol was not specifically powered for the comparison of abediterol with indacaterol, this could be viewed as a potential study limitation. However, the observed treatment differences were >100 mL for the abediterol 5 and 10 μg doses, and 92 mL for the 2.5 μg dose, and all were statistically significant, suggesting that these doses of abediterol were superior to indacaterol in this study.

## Conclusion

Single doses of abediterol (0.625–10 μg) administered via the Genuair® inhaler provided statistically significant and clinically relevant improvements in bronchodilation compared with placebo in patients with moderate to severe COPD. Abediterol 2.5, 5 and 10 μg provided significantly greater improvements in bronchodilation in these patients over 36 h compared with indacaterol 150 μg. Abediterol had low systemic exposure and showed a good overall safety and tolerability profile at all doses. The results suggest that abediterol is a promising new once-daily LABA for the treatment of COPD.

## Abbreviations

AE, adverse event; ANCOVA, analysis of covariance; AUC, area under the curve; AUC_0-12_, area under the curve over the 12 h period immediately after morning IMP administration; AUC_0-24_, area under the curve over the 24 h period immediately after morning IMP administration; AUC_0-∞_, area under the plasma concentration-time curve from zero to infinity; AUC_0-t_, area under the plasma concentration-time curve from zero to the last quantifiable time point; AUC_12-24_, area under the curve over the 12 h nighttime period immediately after morning IMP administration; BID, twice daily; BMI, body mass index; CL/f, total body clearance from plasma; C_max_, maximum plasma concentration; COPD, chronic obstructive pulmonary disease; ERT, eResearch Technology; FEV_1_, forced expiratory volume in 1 s; FVC, forced vital capacity; GOLD, Global initiative for chronic Obstructive Lung Disease; IC, inspiratory capacity; ICS, inhaled corticosteroids; IMP, investigational medicinal product; ITT, intent-to-treat; LABA, long-acting β_2_-agonist; LAMA, long-acting muscarinic antagonist; LS, least squares; PK, pharmacokinetics; QD, once daily; SABA, short-acting β_2_-agonist; SAMA, short-acting muscarinic antagonist; SD, standard deviation; t_1/2_, terminal elimination half-life; TEAE, treatment-emergent adverse event; t_max_, time to reach maximum plasma concentration; Vz/f, apparent volume of distribution
